# An innovative RPE-based approach using machine learning to analyse starter and substitute training load in soccer

**DOI:** 10.5114/biolsport.2026.162046

**Published:** 2026-06-10

**Authors:** Francesco Laterza, Karim Chamari, Giuseppe Caminiti, Giuseppe Annino, Marco Beato, Antonio Bovenzi, Rosario D’Onofrio, Vincenzo Manzi

**Affiliations:** 1 Department of Neurosciences, Biomedicine and Movement Sciences, University of Verona, Verona, Italy; 2 Department of Education and Sport Sciences, Pegaso Telematic University, 80143 Naples, Italy; 3 Higher Institute of Sport and Physical Education of Ksar-Said, University of La Manouba, Manouba, Tunisia; 4 Naufar Center, Doha, Qatar; 5 Department of Human Science and Promotion of Quality of Life, San Raffaele Open University, 00166 Rome, Italy; 6 Cardiology Rehabilitation Unit, IRCCS San Raffaele, Rome, Italy; 7 Department of Systems Medicine, University of Rome Tor Vergata, Roma, Italy; 8 Department of Performance and Sport Science, Hellas Verona, Verona, Italy; 9 Pisa Sporting Club, Pisa, Italy

**Keywords:** Session RPE, Compensatory training, K-means clustering, Training load, Football

## Abstract

Current methods to distinguish starters from substitutes are typically based on playing time. Not considering the physical demands and internal load of different positions can lead to ineffective training and recovery protocols. The aim of this study was to examine whether a k-means clustering approach applied to session-RPE can generate role-specific thresholds that meaningfully differentiate match load profiles between starters and substitutes. We analysed 1,450 player-matches from four professional Italian Serie A teams, using video match analysis to measure total distance (TD) and high-intensity activities: metabolic power events (MPE), high-speed running (HSR), and sprint running (SR). Players were divided based on the role as follows: forwards (FWs), midfielders (MFs), full-backs (FBs), and centre-backs (CBs). Individualized sRPE zones (low, medium, high) were identified with the K-means clustering approach discriminating starters from substituted. FWs, MFs, and FBs were considered substituted, and compensatory training was recommended when the sRPE was within the medium sRPE zone or lower (FWs ≤ 695 a.u., MFs ≤ 711 a.u., and FBs ≤ 726 a.u.). Compensatory training particularly focused on SR was recommended at sRPE = low for FWs (≤ 326.1 a.u.), and at a sRPE ≤ medium for MFs (≤ 711 a.u.). CBs were defined as starters when reporting sRPE values ≥ medium sRPE (> 446 a.u.), and SR training was always recommended. The proposed sRPE-based k-means approach distinguishes fatigued from non-fatigued players, guiding decisions about who should prioritise recovery. Role-specific sprint recommendations help coaches provide appropriate high-velocity exposure to prevent hamstring injuries.

## INTRODUCTION

Substitutions in soccer may alter team tactics, with offensive substitutions enhancing goal-scoring opportunities and defensive ones bolstering defensive effectiveness [1]. Additionally, substitutions are fundamental to mitigate the effect of fatigue on the team and hence to maintain the teams’ physical performance during the match [2]. Since 2020, due to the COVID-19 pandemic, Fédération Internationale de Football Association (FIFA) has adopted the 5-substitution rule, leading teams to perform more substitutions (i.e., 5 vs. 3) compared to the past [3]. This change has provided coaches with more flexibility in managing players’ training load, emphasizing the need to closely monitor and differentiate the physical demands placed on starters and substitutes during matches [4].

It is well known that the match is the most important event of a player’s weekly schedule. The match represents an essential physical, physiological and psychological stimulus for soccer players [5]. Hence, it is important to conduct match analysis and understand how the match load differs for starters and substitutes. Anderson et al. [6] found a clear disparity in high-speed running between starters and their less frequently match-solicited teammates, suggesting that substitutes may not experience the same intensity and volume of physical exertion compared to the starters who played for the entire match duration [4]. Moreover, Anderson et al. [6] also reported that the high-speed running distance of the entire week comes mostly from the match stimulus, underlining the match importance even more. Furthermore, Moreno Pérez and colleagues [7] reported that players with < less than 45 minutes of play in the last two games and with insufficient high-speed running (< 300 m at speed > 24 km/h) in the preceding two matches had a higher incidence of hamstring injuries, shedding light on how the match stimulus may also contribute to injury prevention. In previous research, authors used some arbitrary cut-off to differentiate between starters and non-starters; for instance, some consider starters players who competed for more than 60 minutes, and substitutes as those who played less than 60 minutes [8–10]. However, other authors used different cut-off [6, 11], which may lead to ambiguity and potentially inconsistencies in training and recovery protocols. Additionally, the match-time-play method does not consider players’ external or internal load, or the physical performance differences across roles [12–15]. Therefore, the match-time play method may be suboptimal for training purposes following a football match.

An alternative method is therefore necessary to address some of these issues. Clustering is a useful tool in data science that allows a cluster structure to be found in a data set based on the greatest similarity within the same cluster and the greatest dissimilarity between different clusters [16]. Specifically, K-means clustering is a widely used unsupervised machine learning algorithm for partitioning data into distinct groups based on similarity [17, 18]. K-means clustering has been applied to classify athletes or performance metrics into clusters, allowing researchers to identify patterns or trends within a dataset [19], overcoming the limitation of arbitrary gathering approach. This machine learning approach could be used to classify players based on their session-RPE and to provide with session-RPE thresholds to discriminate starters from substitutes. The session-RPE (sRPE) determined by multiplying the training duration in minutes by the RPE given for the entire session, as described by Foster et al. [20], has been found to be a good indicator of global internal load in soccer (sRPE = RPE ´ time) [21]. Its effectiveness as a comprehensive measure of training load in elite soccer has been well documented [22, 23]. This method allows for a more nuanced assessment of player load, considering not just the duration of play but also the perceived intensity of effort, which is crucial for understanding and managing player fatigue and performance [23, 24].

The aim of this study was to examine whether a k-means clustering approach applied to session-RPE can generate role-specific thresholds that meaningfully differentiate match load profiles between starters and substitutes. By this approach, we hope to provide a more accurate and nuanced understanding of players’ match loads, facilitating better training and recovery strategies for soccer teams.

## MATERIALS AND METHODS

### Experimental approach to the problem

This is a retrospective observational analysis of existing data aimed at creating role-dependent sRPE thresholds to distinguish starters from substitutes. Consequently, the first step of this study was to develop thresholds of session-RPE. The session-RPE was calculated by multiplying the RPE score by the match duration in minutes, as described by Foster et al. [20]. The RPE was collected using Borg’s CR-10 scale [25], administered 30 minutes after the end of each match. Preliminary k-means clustering was applied to the session-RPE data for each role, with the optimal number of clusters determined using the elbow method. As only one variable was included, standardization was not applied because scaling would not affect Euclidean distances or cluster assignment in one-dimensional k-means clustering. By evaluating the within-cluster sum of squares across a range of cluster counts, the “elbow” point [26], where additional clusters yielded only marginal improvements, was identified, as shown in Figure 1. This data-driven approach established objective cut-off values for categorizing session-RPE into low, medium, and high. The locomotor activity in the high session-RPE zone was considered as the complete match stimulus. The second step was to assess whether statistical differences in locomotor activity were detected among different sRPE thresholds. This approach helped to determine the sRPE cut-off below which players should be considered substitutes.

**FIG. 1:**
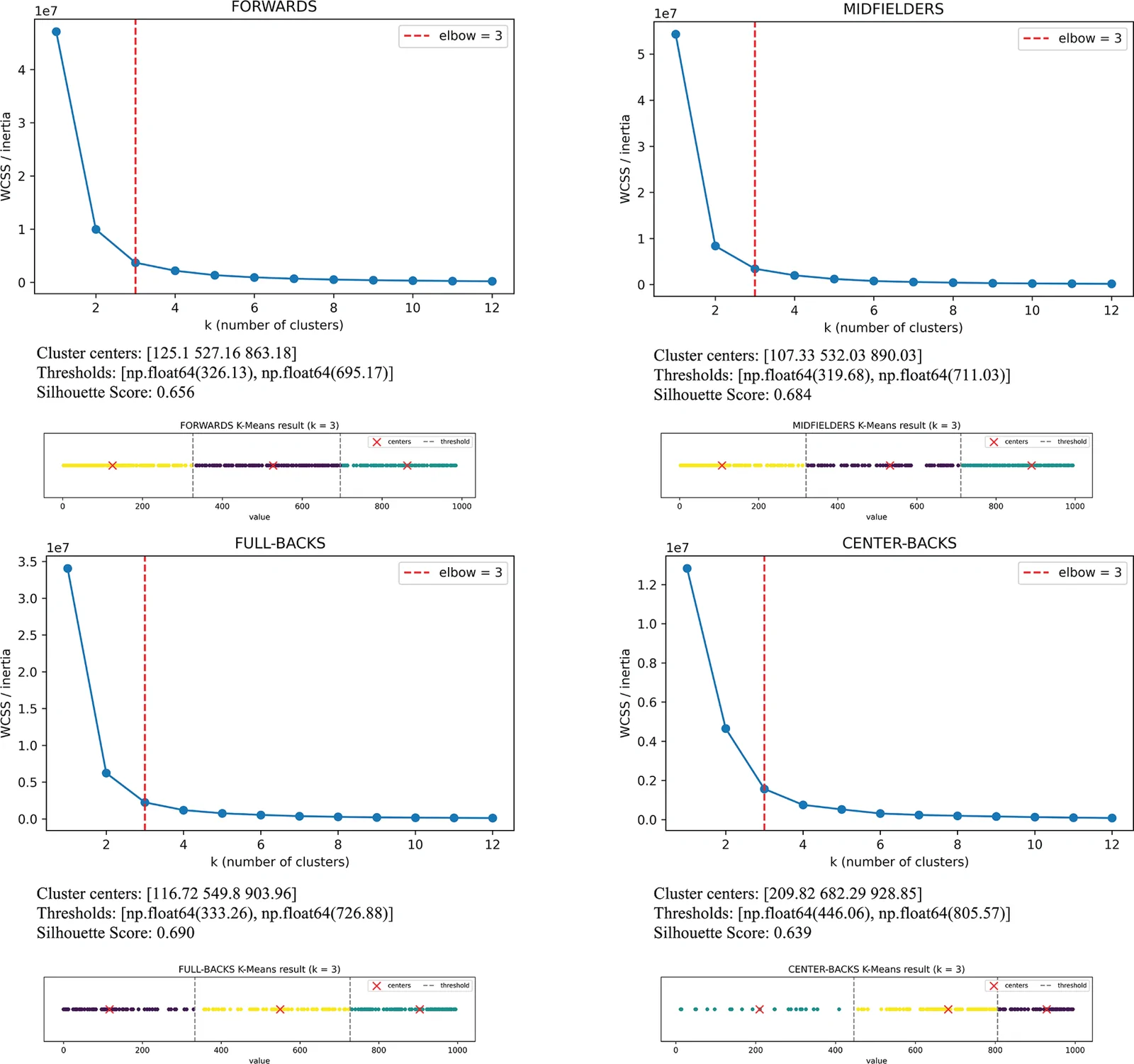
Elbow method plots and K-means clustering results for Midfielders, Forwards, Center-Backs, and Full-Backs. The optimal number of clusters (k = 3) was identified at the elbow point (dashed red line). Cluster assignments are shown with centroids (red ×) and decision thresholds (gray dashed lines).

Four distinct seasons of four professional teams competing in the Italian First Division (Serie A) were analysed. Ninety-five official matches between the 2017 and 2022 seasons were analysed using video match analysis. All the teams competed with the 4-3-3 playing formation. When analysing the locomotor activity of soccer players, it is crucial to recognize the association between high-intensity activities and the most decisive soccer game events, because they are linked to scoring and repossession of the ball [27]. Consequently, the kinematic variables considered were number of metabolic power events (MPE) [28], high-speed running distance (HSR) (19 km/h ≤ v < 25 km/h), sprint running distance (SR) (v ≥ 25 km/h), and the distance (m) covered at ≥ 25 W/kg (D25) [28]. The total distance (TD) covered was also reported. The locomotor activity data were collected using the validated multicamera video analysis system STATS SportVU (STATS LLC, Chicago, IL, U.S.A.) [29]. Finally, the players were divided based on the role as follows: forwards (FWs), midfielders (MFs), full-backs (FBs), and centre-backs (CBs); goalkeepers were not considered in this investigation. Data of players who played extra time were discarded.

### Participants

All the players had at least five years of competitive experience in the Italian First Division. The descriptive statistics of participants are reported in Table 1. They were monitored over multiple seasons (11 months each), including pre-season, in-season, and all official matches (Serie A and Italian Cup matches). All participants were informed about the study protocol and gave their informed consent to participate. This study was approved by the Internal Research Board of the University of San Raffaele. All the procedures involved in this study were in accordance with the Declaration of Helsinki.

**TABLE 1:** Descriptive statistic of participants, data reported as mean and standard deviations.

Variables	Forwards (n = 25)	Midfielders (n = 20)	Full-backs (n = 18)	Center-backs (n = 17)
Age (years)	25.7 ± 4.9	25.0 ± 4.5	25.7 ± 2.3	27.1 ± 2.8
Height (cm)	180.1 ± 4.8	180.9 ± 3.7	181.4 ± 2.7	189.0 ± 3.7
Body weight (kg)	77.9 ± 2.8	77.0 ± 4	79.9 ± 2.7	85.1 ± 4.0
Body fat (%)	11.4 ± 2.5	10.5 ± 1.9	11.3 ± 1.7	10.9 ± 2.1

### Video match analysis

Match analysis was performed using a STATS SportVU multicamera video analysis system (STATS LLC, Chicago, IL, U.S.A.), tracking at 25 Hz rates. Previous validation reported a typical error of 2.7% for total distance [29]. Raw data were provided via cartesian coordinates by K-Sport (primary data have been smoothed at 5 Hz). The STATS SportVU tracking system transports the data of performance by extracting and processing coordinates of players (X, Y) and the ball (X, Y, Z) through HD cameras as well as specific software and statistical algorithms [29]. Player movements were captured during matches through fixed cameras located at the stadium roof level. The data were analysed by two sources, i.e. STATS Viewer and STATS Dynamix, both provided by STATS Perform, to create a dataset on each player’s physical and technical performance. Data extracted from STATS SportVU were downloaded and analysed using the software gpexe (https://www.gpexe.com).

### Statistical analysis

The data sorting was carried out using Python programming language (version 17.0.9). The open-source libraries implemented for k-means clustering were NumPy, Sklearn.cluster and Matplotlib.pyplot. Machine learning k-means cluster analysis theory and application were recently described in detail [17]. The linear mixed model is applicable to repeated-measures data from unbalanced designs. This approach was suitable for the current study, as players varied in the number of matches they participated in and the sRPE zones they were in [12]. Mixed linear modelling can also cope with the mixture of both fixed and random effects, as well as missing data from players [30]. In the current study, linear mixed-effect models were fitted using restricted maximum likelihood (REML) estimation, with sRPE zones (low, medium, and high) treated as a categorical fixed effect, and individual player IDs as a random intercept to account for repeated measures within players. Post-hoc test comparisons with Bonferroni correction were applied when a statistical difference was detected. Statistical significance was set at p < 0.05. The estimated differences were divided by the estimated between player standard deviations to calculate the effect size (ES). Effect sizes were calculated from the *t* and *df* of the contrast and interpreted using Cohen’s d principle as follows: ES ≤ 0.2 trivial, 0.2 < ES ≤ 0.6 small, 0.6 < ES ≤ 1.2 moderate, 1.2 < ES ≤ 2.0 large, 2.0 < ES ≤ 4.0 very large, and ES > 4.0 extremely large [31]. Statistical analysis was carried out with the software Jamovi (version 2.3.18.0). Full model diagnostics including fit indices (AIC, BIC), marginal and conditional R-squared values, and intraclass correlation coefficients for all models are reported in Supplementary Table S1.

## RESULTS

Fifty-two match data did not meet the inclusion criteria and were consequently excluded. We analysed a total of 1,450 player-matches, which comprised 459 FWs, 434 MFs, 297 FBs and 260 CBs.

Elbow method plots and K-means clustering results for midfielders, forwards, centre-backs, and full-backs are shown in Figure 1. The individualized sRPE thresholds for each role, as well as the average values for each kinematic variable associated with roles and session-RPE zones, are reported in Table 2.

**TABLE 2:** Individualized sRPE Thresholds and Average Kinematic Variable Values by Role and Session-RPE zones.

	sRPE zone	Time played (min)	sRPE thresholds (a.u.)	sRPE (a.u.) ± SD	sRPE (a.u.) ± CI 95%	Total Distance (m)	Power Events (n)	D25 (m)	HSR (m)	SR (m)
**FORWARDS**	LOW (n = 186)	29± 14.7	sRPE ≤ 326.1	125.1 ± 82.4	125.1 ± 11.9	3299.2 ± 1694	55.6 ± 29.4	588.9 ± 300.4	235.1 ± 127.5	64.8 ± 47.9

	MEDIUM (n = 138)	71 ± 12.1	326.1 < sRPE ≤ 695.2	527.2 ± 102.3	527.2 ± 17.2	8053.4 ± 1319.8	129 ± 25.1	1389.7 ± 319.3	543.1 ± 153.8	168 ± 94.6

	HIGH (n = 135)	93 ± 5.9	sRPE > 695.2	863.2 ± 87	863.2 ± 14.8	10232.7 ± 871.4	163.2 ± 23.9	1668.4 ± 286.2	685.1 ± 160.7	204.2 ± 94.2

**MIDFIELDERS**	LOW (n = 139)	25 ± 15	sRPE ≤ 319.7	107.3 ± 85.6	107.3 ± 14.3	3007.9 ± 1756.2	54.4 ± 31.7	592.9 ± 346	226 ± 135.7	43.2 ± 39.2

	MEDIUM (n = 90)	74 ± 14.8	319.7 < sRPE ≤ 711	532 ± 115.3	532 ± 24.1	8650 ± 1607.3	151.7 ± 29.8	1661.7 ± 430.3	540.1 ± 181	106 ± 55.6

	HIGH (n = 205)	94 ± 4.5	sRPE > 711	890 ± 78.3	890 ± 10.8	11033.5 ± 789.9	183.2 ± 28.4	1915.8 ± 387.1	780.9 ± 229.5	136.1 ± 68.3

**FULL-BACKS**	LOW (n = 75)	26 ± 14.5	sRPE ≤ 333.2	116.7 ± 89.2	116.7 ± 20.5	2971.4 ± 1667	48.2 ± 27.2	614.9 ± 401	218.3 ± 133.1	87.2 ± 70.8

	MEDIUM (n = 67)	74 ± 13.2	333.2 < sRPE ≤ 726.9	549.8 ± 108.3	549.8 ± 26.4	8205.5 ± 1359.8	133.2 ± 26.6	1555.6 ± 390.5	543.4 ± 143.3	194.7 ± 104.3

	HIGH (n = 155)	95 ± 2.3	sRPE > 726.9	904 ± 75.6	904 ± 12.0	10489.3 ± 544.8	166.3 ± 22.1	1876.3 ± 492.1	757.2 ± 155.4	222 ± 97.9

**CENTER-BACKS**	LOW (n = 24)	39 ± 14.7	sRPE ≤ 446	209.8 ± 115.9	209.8 ± 48.9	4186.4 ± 1623.9	66.5 ± 29.6	808.8 ± 389.8	202.2 ± 82.9	62.4 ± 55

	MEDIUM (n = 120)	94 ± 6.8	446 < sRPE ≤ 805.5	682.3 ± 89.7	682.3 ± 16.2	9817.2 ± 839.2	165.2 ± 33.3	1516.9 ± 291.1	477.9 ± 170.6	108.5 ± 54.4

	HIGH (n = 116)	95 ± 2.5	sRPE > 805.5	928.8 ± 51	928.8 ± 9.4	10144.6 ± 579.8	157.2 ± 24	1574.1 ± 345	558.3 ± 145.7	119.6 ± 61.6

Results presented as average ± standard deviation. D25 = distance (m) covered at ≥ 25 watt/kg, HSR = distance covered at 19 km/h ≤ v < 25 km/h, SR = distance covered at v ≥ 25 km/h, n = number of players.

Statistical differences were detected when kinematic variables of different sRPE zones were compared. The results of the statistical analysis are presented in Figures 2–5.

**FIG. 2:**
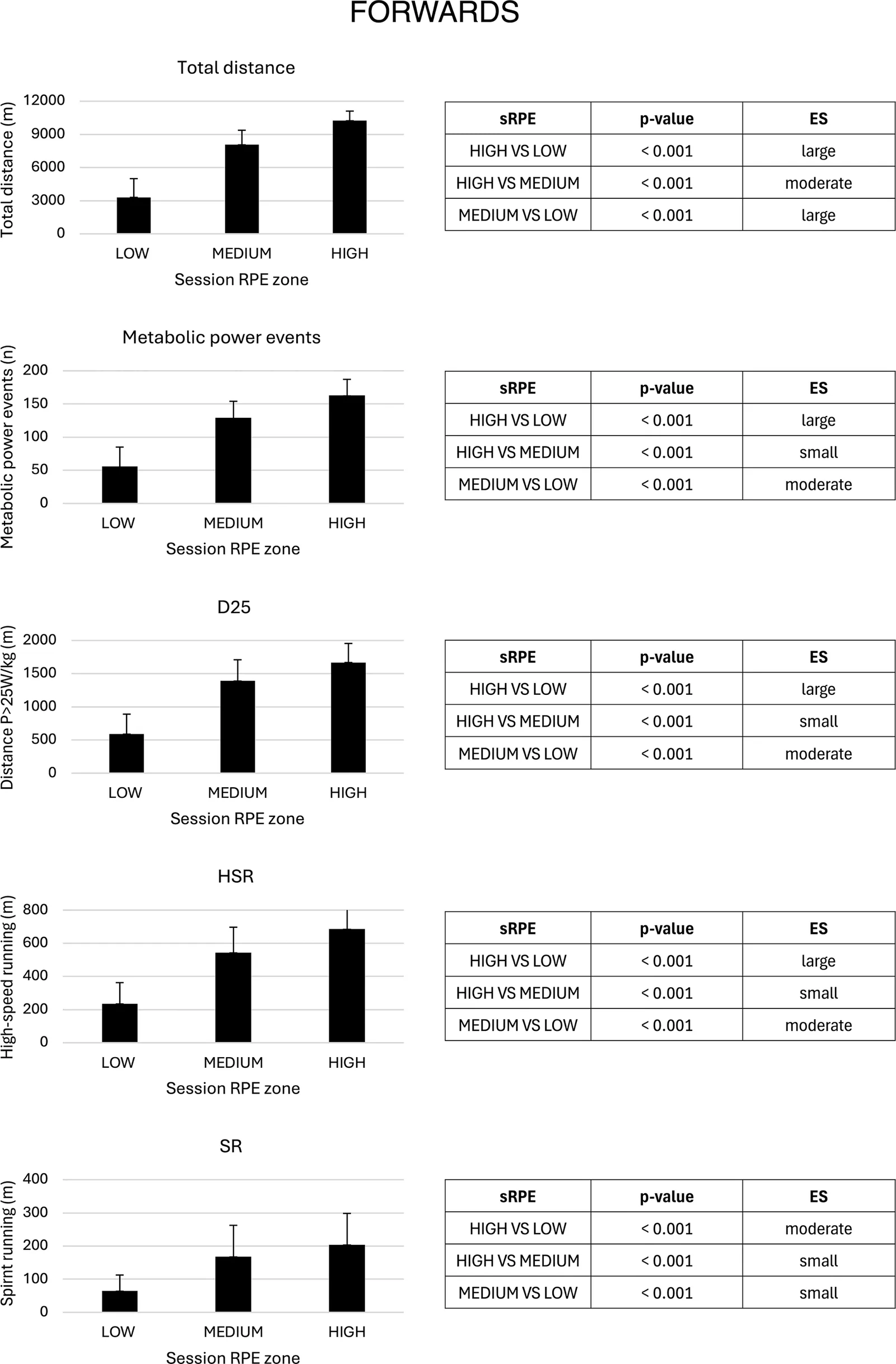
Comparison of the locomotor activity variables across different session-RPE zones in Forwards. ES=effect size, ES ≤ 0.2 = trivial, 0.2< ES ≤ 0.6 = small, 0.6 < ES ≤ 1.2 = moderate, 1.2 < ES ≤ 2.0 = large, 2.0 < ES ≤ 4.0 = very large, and ES > 4.0 = extremely large. HSR = High-Speed Running distance, SR = Sprint running distance (m), D25= distance (m) covered at ≥ 25watt/kg. The error bars represents the SD.

**FIG. 3:**
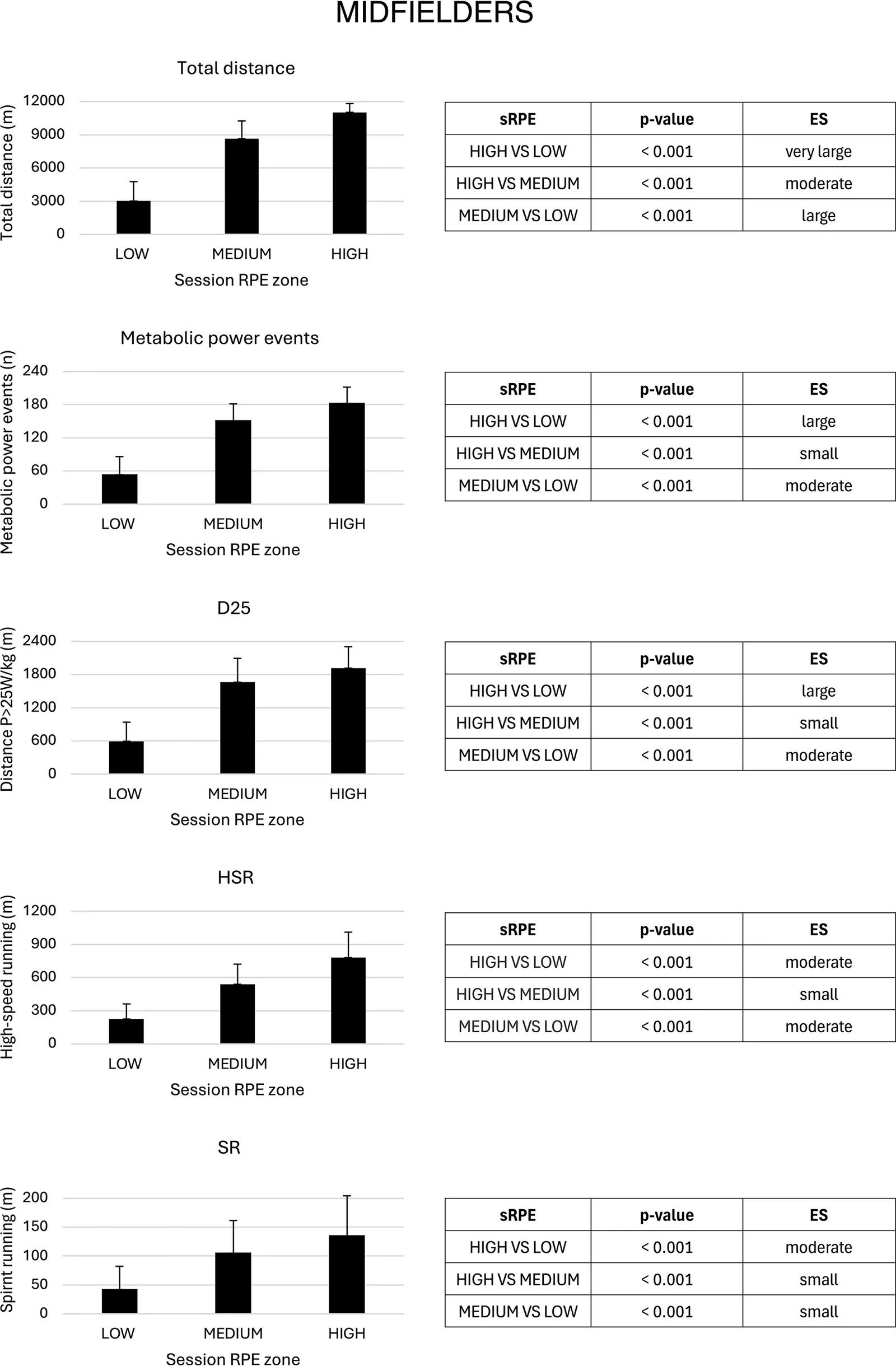
Comparison of the locomotor activity variables across different session-RPE zones in Midfielders. ES=effect size, ES ≤ 0.2 = trivial, 0.2< ES ≤ 0.6 = small, 0.6 < ES ≤ 1.2 = moderate, 1.2 < ES ≤ 2.0 = large, 2.0 < ES ≤ 4.0 = very large, and ES > 4.0 = extremely large. HSR = High-Speed Running distance, SR = Sprint running distance (m), D25= distance (m) covered at ≥ 25watt/kg. The error bars represents the SD.

**FIG. 4:**
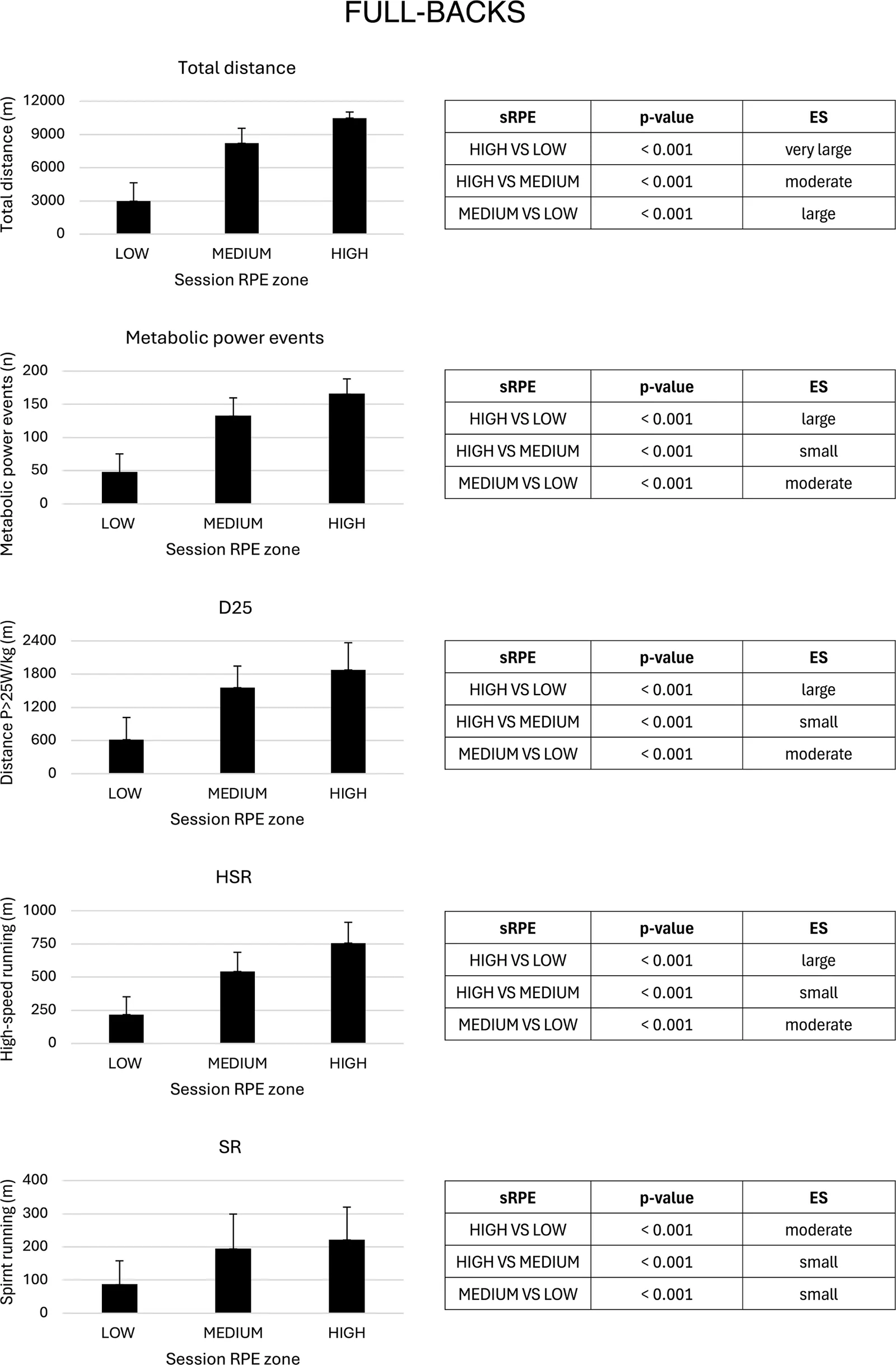
Comparison of the locomotor activity variables across different session-RPE zones in Full-backs. ES=effect size, ES ≤ 0.2 = trivial, 0.2< ES ≤ 0.6 = small, 0.6 < ES ≤ 1.2 = moderate, 1.2 < ES ≤ 2.0 = large, 2.0 < ES ≤ 4.0 = very large, and ES > 4.0 = extremely large. HSR = High-Speed Running distance, SR = Sprint running distance (m), D25= distance (m) covered at ≥ 25watt/kg. The error bars represents the SD.

**FIG. 5:**
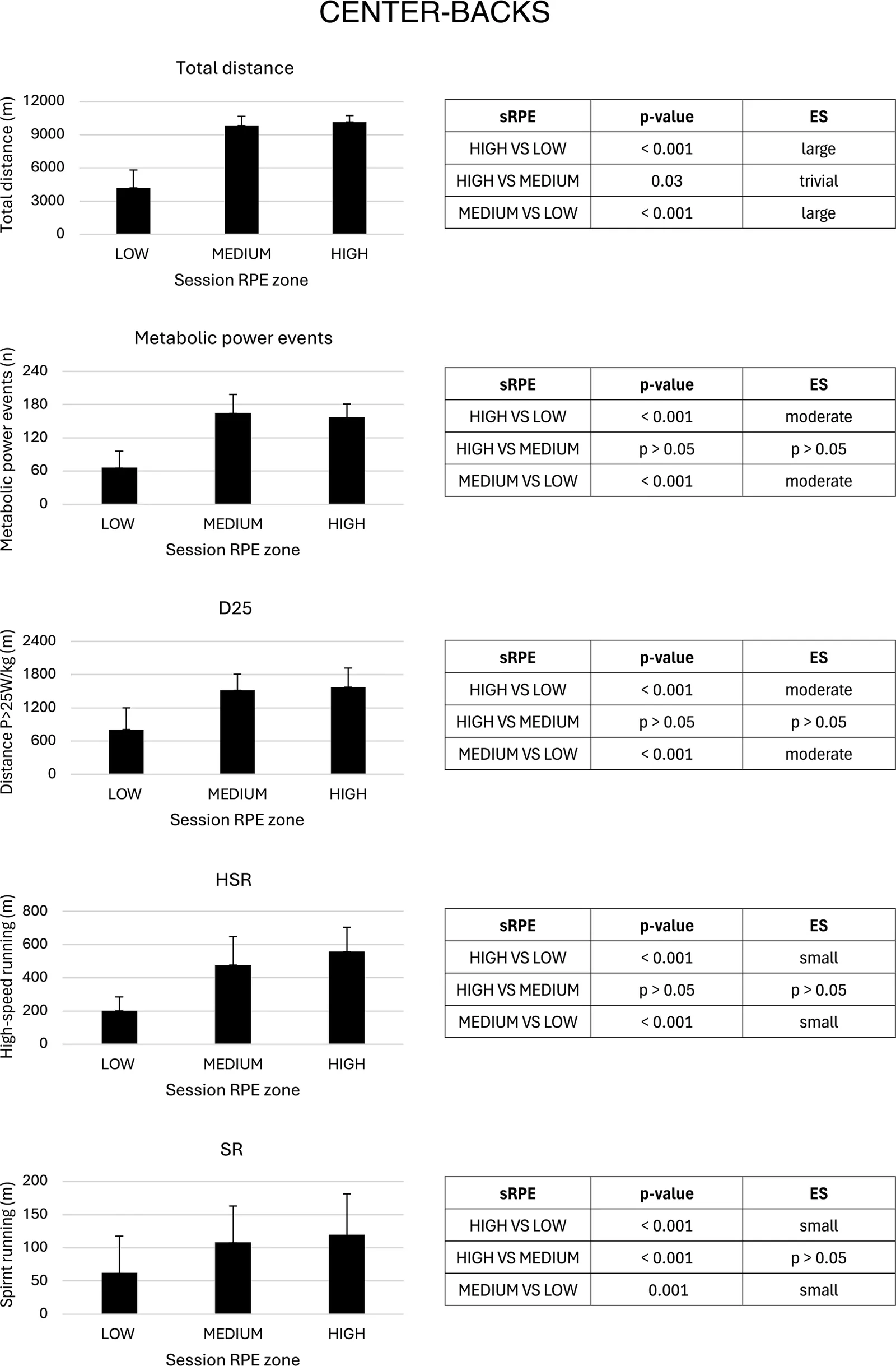
Comparison of the locomotor activity variables across different session-RPE zones in Center-Backs. ES=effect size, ES ≤ 0.2 = trivial, 0.2< ES ≤ 0.6 = small, 0.6 < ES ≤ 1.2 = moderate, 1.2 < ES ≤ 2.0 = large, 2.0 < ES ≤ 4.0 = very large, and ES > 4.0 = extremely large. HSR = High-Speed Running distance, SR = Sprint running distance (m), D25= distance (m) covered at ≥ 25watt/kg. The error bars represents the SD.

Forwards, midfielders and full-backs reported statistical differences among all the locomotor activity variables in different sRPE zones. Centre-backs also reported statistical differences among all the locomotor activity variables in different sRPE zones, with few exceptions. No statistical difference was detected between high and medium sRPE for metabolic power events, D25, HSR and SR.

## DISCUSSION

The aim of this study was to create a new sRPE-based approach to analyse the training load of starters and substitutes using a machine learning k-means cluster analysis and considering role, playing time, internal load, and external load. Although studies have applied the k-means approach to identify thresholds of external workload demands [32] or to determine the ball possession of soccer teams [19], this is the first study to apply the k-means clustering approach to identify different session-RPE zones. Forwards, midfielders, and fullbacks exhibited significantly different values for TD, MPE, HSR, D25, and SR at high sRPE compared to medium and low sRPE. In contrast, centre-backs did not show significant differences in MPE, D25, HSR, and SR between high and medium sRPE, although significant differences were observed in the other sRPE comparisons for all analysed variables.

Table 2 shows the different sRPE thresholds, individualized through the machine learning approach with the respective locomotor activity associated; in Figures 2–5 a graphical representation and the statistical differences detected are provided. As shown, the locomotor activity of FWs, MFs and FBs who report a session-RPE in the medium range (or lower) is significantly lower in all of the kinematic variables analysed compared to those who received the complete match stimulus, thus indicating the medium sRPE zone as the ideal one to discriminate starters from substitutes. The same reasoning applies to CBs, who exhibit a low session-RPE as the discriminant one. Given that the greatest part of the distance covered at sprint speed (> 25 km/h) comes from the match [6, 33], probably due to training in a small part of the pitch—i.e., small sided games [34]—the analysis of the SR requires careful examination. Straight sprinting, defined as a very-high-intensity run with (near) maximal velocity, was found to be the most frequent action in goal situations in professional football [35]. In addition, hamstring injuries usually occur at > 25 km/h and at > 80% of the maximal velocity [36, 37]; therefore, the results for training volume of this capacity of performing high-intensity runs are extremely important. FWs and MFs, as reported in Table 2, covered on average 168 ± 94.6 m and 106 ± 55.6 m at SR in the medium sRPE zone, respectively. In light of Moreno Pérez and colleagues’ findings [7], who reported that players with insufficient high-speed running (< 300 m) in the preceding two matches had a higher incidence of hamstring injuries, prescribing compensatory sprint-focused training is particularly important for forwards when low session-RPE is reported, whereas midfielders might benefit from top-up training with a greater focus on sprint running even at the medium sRPE level.

Full-backs in the medium session-RPE zone covered an average of 194.7 ± 104.3 m of sprint-running distance, a value that closely mirrors that of forwards in the high sRPE zone and exceeds that of midfielders falling in the high sRPE zone. This observation supports the literature indicating that, within the 4-3-3 formation, full-backs exhibit the highest sprint-running demands [14]. Given their elevated SR activity, the focus of compensatory training for fullbacks should shift from injury prevention to providing a proper stimulus that compensates for the missing locomotor activities and helps maintain performance throughout the season. Consequently, fullbacks reporting medium session-RPE may benefit from a compensatory training regimen that targets all the analysed kinematic variables—total distance, MPE, D25, HSR, and SR—to comprehensively compensate for the locomotor demands not met during matches.

CBs reported a different pattern suggesting that that players need compensatory training only when they report a low sRPE. However, it can be noted (Table 2) that the SR distance covered by CBs is not sufficient to cover the 300 m in the past two matches as recommended by Pérez et al. [7]. Consequently, training at SR may be beneficial even though players have already achieved the proper match training stimulus. Figure 6 sums up our proposed strategy.

**FIG. 6:**
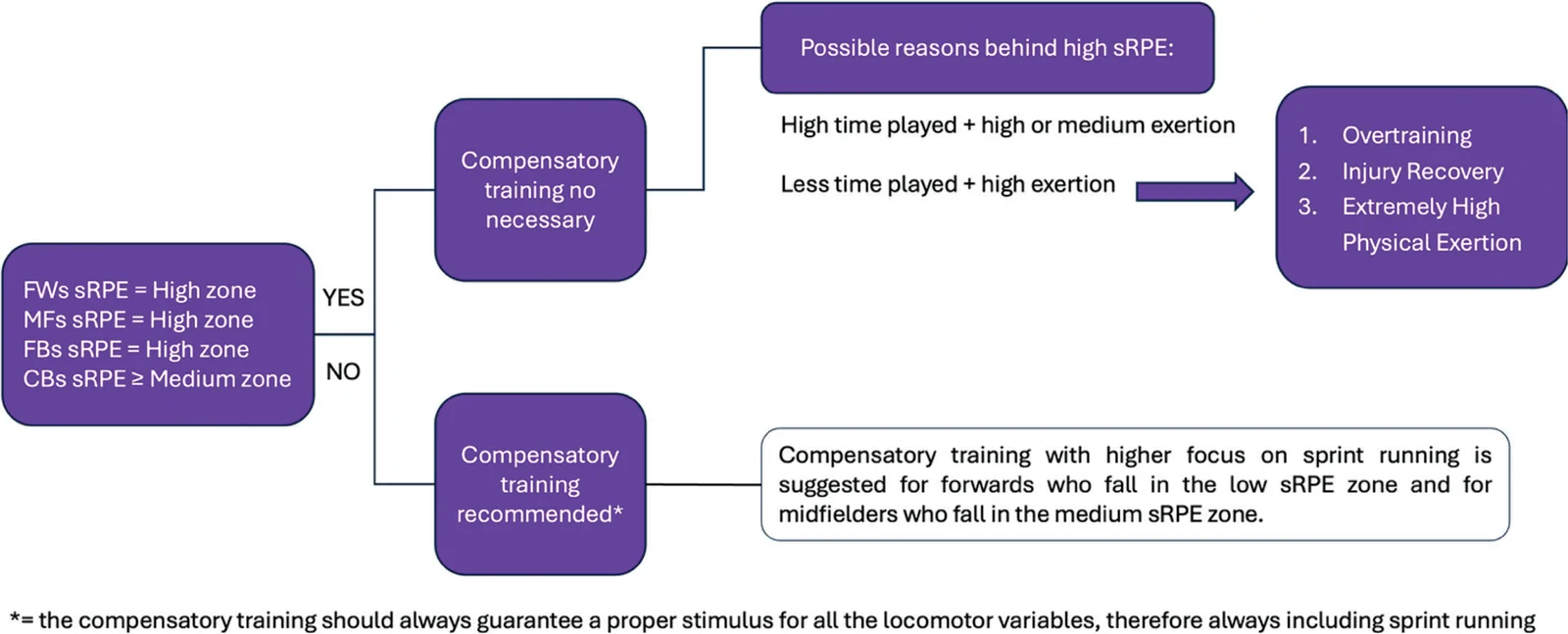
Recommended strategy from authors. FWs=forwards, MFs=midfielders, FBs=full-backs, and CBs= center-backs.

The following process is what the authors recommend. A midfielder who reports 630 a.u. of sRPE falls in the medium sRPE zone (see Table 2). Consequently, compensatory training is necessary with a greater focus on SR (see Figure 6), since the sprint running distance in the medium sRPE zone is not sufficient to guarantee an exposure that will prevent hamstring injuries (see Table 2). The proposed method integrates internal load, player role, and playing time, enabling the identification of individualized thresholds specific to the team’s needs. Although in this investigation the 4-3-3 playing formation was considered, further research can easily apply this rationale across various team formations and playing styles. Coaches can apply this rationale to teams using other formations and adjust for the unique playing style of individual players. Moreover, this approach may allow players who report a high level of perceived exertion (due to greater locomotor activity [21], a state of non-functional over-reaching [38], or a lower level of conditioning in the case of players who have been injured and are returning to play) to be considered as a starter. Likewise, players who received an insufficient training stimulus due to low locomotor activity exerted during the match, detected through a low reported sRPE, may be eligible to receive compensatory training.

To enhance the generalizability of the method, situational variables such as ball possession, opponents’ level and match location (home or away) were not taken into consideration, as their inclusion could potentially hinder the practical application of the methodology in real-world settings.

It is recommended that the interpretation of results also consider each locomotor variable, which would facilitate a more effective prescription of compensatory training programmes.

Since the derived thresholds are specific to elite players, further research should investigate the applicability of this method in youth categories given the easy implementation of load monitoring through session-RPE [39]. Although the session-RPE thresholds presented in this study could help identify starters and substitutes without requiring advanced tracking technologies during matches, it should be acknowledged that GPS or equivalent tracking systems would still be necessary to guide and monitor the compensatory training loads. In settings where such technologies are unavailable, future studies should explore alternative approaches to prescribe compensatory loads based on internal load indicators alone.

### Limitations and future developments

Some limitations should be noted when interpreting the findings of this study. A direct comparison between the sRPE-based thresholds and traditional minute-based classifications was not performed and warrants future investigation. The k-means clustering was applied to the entire dataset to maximize the precision of the derived sRPE thresholds. While this approach does not allow for internal validation procedures (e.g., cross-validation), it was chosen to establish thresholds based on the largest possible sample. External validation using independent datasets from other teams or leagues is warranted to confirm the generalizability of these thresholds. The playing formation analysed in this study was 4-3-3. Therefore, readers should be mindful when using the thresholds of session-RPE reported for other playing modules, given the evidence of how the formation and the tactical behaviour of the team affect the locomotor activity of soccer players [19, 40]. Additionally, accelerations were not included in this analysis due to varying thresholds used by teams (two teams used 2.5 m · s^−2^, while the other two used 3 m · s^−2^). The year of the seasons analysed differed among teams, and slight variations in the play’s requirements could have affected the performance. Moreover, the re-warm-up strategy was neither considered nor standardized across teams, which could have led to variations in performance output after half-time due to differing re-warm-up protocols [41]. Further research studies where the proposed approach is implemented are needed to confirm its usefulness and to address the above-mentioned limitations.

## CONCLUSIONS

This study introduces a novel approach using session-RPE (sRPE) and machine learning k-means cluster analysis to differentiate between players who required additional training following a match in professional soccer, accounting for role-specific demands, playing time, and internal load. Our study underscores the limitations of traditional time-based methods and highlight the significance of incorporating positional roles, sRPE and external training load in distinguishing players’ match load. Our findings suggest that for forwards, midfielders, and full-backs, the high sRPE threshold can effectively serve as a criterion to identify starters, except when considering SR. Compensatory training focused on this capacity is recommended for FWs and MFs, who fall into the low and very low sRPE zones. FBs, characterized by consistently high SR across all sRPE zones, should focus their compensatory training exclusively on the other missing locomotor activities unless the sRPE falls into the very low sRPE zone, where the compensatory training of SR is suggested. Conversely, centre-backs displayed a distinct pattern with minimal differences in SR across all sRPE zones, reinforcing the need for tailored compensatory training that addresses their unique role demands. The individualized sRPE thresholds and corresponding recommendations provide a comprehensive guide for coaches and will hopefully contribute to facilitating the decision-making process in the management of starters and substitutes.

## Supplementary Material

An innovative RPE-based approach using machine learning to analyse starter and substitute training load in soccer
