# Supplementary material for: An innovative RPE-based approach using machine learning to analyse starter and substitute training load in soccer
Source: Biol Sport. 2026 Jun 10;43:1473–85. doi: 10.5114/biolsport.2026.162046 (PMC13617481; doi:10.5114/biolsport.2026.162046)
Supplement: Supplementary file 1 [file JBS-43-58107-s1.pdf]

SUPPLEMENTARY MATERIALS

SUPPLEMENTARY TABLE S1. Linear Mixed Model Fit Statistics by Playing Position and Outcome Variable

Position	Outcome Variable	n (obs)	n (athletes)	AIC	BIC	Log-Likelihood
CB	Distance (m)	260	17	4233.35	4216.09	-2094.14
CB	Power Events	260	17	2374.24	2377.43	-1174.81
CB	D25	260	17	3607.68	3595.62	-1783.91
CB	HSR (m)	260	17	3236.01	3229.17	-1600.68
CB	SR (m)	260	17	2849.27	2848.62	-1410.41
FB	Distance (m)	297	18	5023.91	5007.96	-2489.74
FB	Power Events	297	18	2726.26	2732.28	-1351.91
FB	D25	297	18	4277.58	4265.64	-2118.58
FB	HSR (m)	297	18	3782.13	3777.11	-1874.32
FB	SR (m)	297	18	3471.03	3468.66	-1720.09
MF	Distance (m)	434	20	7437.04	7421.46	-3695.55
MF	D25	434	20	6237.01	6228.34	-3098.99
MF	Power Events	434	20	4133.29	4140.44	-2055.04
MF	HSR (m)	434	20	5659.43	5655.56	-2812.6
MF	SR (m)	434	20	4724.84	4728.34	-2348.99
FW	Distance (m)	459	25	7857.98	7842.99	-3906.17
FW	Power Events	459	25	4224.3	4232.99	-2101.17
FW	D25	459	25	6429.02	6422.53	-3195.94
FW	HSR (m)	459	25	5754.77	5753.25	-2861.3
FW	SR (m)	459	25	5180.37	5182.49	-2575.92

Position	R^2 Marginal	R^2 Conditional	ICC	Variance (Athlete)	Variance (Residual)	Converged
CB	0.785	0.824	0.178	134022	617258	Yes
CB	0.43	0.672	0.424	335	455	Yes
CB	0.271	0.797	0.722	125874	48573	Yes
CB	0.197	0.558	0.449	10169	12455	Yes
CB	0.072	0.156	0.09	307	3104	Yes
FB	0.882	0.884	0.015	18887	1250000	Yes
FB	0.796	0.821	0.125	74.6	521.7	Yes
FB	0.452	0.872	0.766	273596	83362	Yes
FB	0.654	0.724	0.203	4548	17869	Yes
FB	0.263	0.509	0.334	3057	6087	Yes
MF	0.859	0.888	0.204	377619	1480000	Yes
MF	0.645	0.849	0.575	117460	86972	Yes
MF	0.774	0.826	0.228	214	726	Yes
MF	0.598	0.735	0.339	12269	23921	Yes
MF	0.348	0.443	0.146	494	2890	Yes
FW	0.805	0.856	0.261	506405	1440000	Yes
FW	0.73	0.805	0.279	202	522	Yes
FW	0.622	0.817	0.516	64983	60855	Yes
FW	0.629	0.754	0.337	7357	14479	Yes
FW	0.343	0.588	0.372	2442	4115	Yes

CB = Center Backs; FB = Full Backs; MF = Midfielders; FW = Forwards, n (obs) = number observation
AIC = Akaike Information Criterion; BIC = Bayesian Information Criterion; ICC = Intraclass Correlation Coefficient
R^2 Marginal = variance explained by fixed effects; R^2 Conditional = variance explained by fixed + random effects
All models were fitted using REML estimation with player ID as random intercept. Optimizer: BOBYQA.
D25= distance (m) covered at ≥ 25 watt/kg HSR = High-Speed Running distance; SR= Sprint Running distance (> 6.94 m/s).
